# Modulation of Vasodilator Response via the Nitric Oxide Pathway after Acute Methyl Mercury Chloride Exposure in Rats

**DOI:** 10.1155/2013/530603

**Published:** 2013-08-19

**Authors:** S. Omanwar, B. Saidullah, K. Ravi, M. Fahim

**Affiliations:** ^1^School of Sciences, Indira Gandhi National Open University, New Delhi 110068, India; ^2^Department of Physiology, V.P. Chest Institute, University of Delhi, New Delhi 110007, India; ^3^Jamia Hamdard University, New Delhi 110062, India

## Abstract

Mercury exposure induces endothelial dysfunction leading to loss of endothelium-dependent vasorelaxation due to decreased nitric oxide (NO) bioavailability via increased oxidative stress. Our aim was to investigate whether acute treatment with methyl mercury chloride changes the endothelium-dependent vasodilator response and to explore the possible mechanisms behind the observed effects. Wistar rats were treated with methyl mercury chloride (5 mg/kg, *po*.). The methyl mercury chloride treatment resulted in an increased aortic vasorelaxant response to acetylcholine (ACh). In methyl-mercury-chloride-exposed rats, the % change in vasorelaxant response of ACh in presence of N_**ω**_-Nitro-L-arginine methyl ester hydrochloride (L-NAME; 10^−4^ M) was significantly increased, and in presence of glybenclamide (10^−5^ M), the response was similar to that of untreated rats, indicating the involvement of NO and not of endothelium-derived hyperpolarizing factor (EDHF). In addition, superoxide dismutase (SOD) + catalase treatment increased the NO modulation of vasodilator response in methyl-mercury-chloride-exposed rats. Our results demonstrate an increase in the vascular reactivity to ACh in aorta of rats acutely exposed to methyl mercury chloride. Methyl mercury chloride induces nitric oxide synthase (NOS) and increases the NO production along with inducing oxidative stress without affecting the EDHF pathway.

## 1. Introduction

Humans have been exposed to mercury for centuries and there is vast data indicating the harmful effects of this heavy metal. The role of mercury toxicity as a possible risk factor in cardiovascular disease has been discussed, and reports on the toxic effects of metals in several diseases among humans including the vascular diseases have been well documented [1–4]. The loss of endothelial function is one of the most commonly observed vascular effects of mercury exposure [5–9]. As has been widely reported, this endothelial dysfunction leads to a loss of endothelium-dependent vasorelaxation. This loss at least in part is due to the reduction in NO bioavailability because of increased ROS production [8, 9]. Previous studies from our group have demonstrated that chronic mercury exposure selectively impairs the NO pathway as a consequence of oxidative stress, while endothelium-dependent hyperpolarizing factor (EDHF) is able to maintain endothelium-dependent relaxation at a reduced level [9]. Though an increase in NO was observed in our previous study, it failed to attain significance as reductions in NO bioavailability may be due to the increased ROS production, which was responsible for the endothelial dysfunction [10, 11]. In contrast, interestingly, the previous study by Golpon et al. has reported that mercury *in vitro* induces an endothelial-dependent vasorelaxation in isolated aortic rings which is totally blocked by the NOS inhibitor L-NAME indicating that mercury *per se* acts through the NO pathway [7].

Therefore, the aim of this study was to assess the effect of acute treatment of methyl mercury chloride on (a) the endothelium-dependent vasodilator responses in rat aorta; (b) ROS and NO participation in these vascular responses; and (c) NO and ROS production.

## 2. Methods

### 2.1. Animals

Wistar rats (250–300 gm) of either sex were maintained according to the recommendations by the National Accreditation Board of Testing and Calibration Laboratories (NABL), and the study was approved by the VP Chest Institute's animal ethical committee. During treatment, rats were housed at a constant room temperature, humidity, and light cycle (12 : 12 h light-dark), with free access to tap water and were fed with standard chow *ad libitum*.

Rats were divided into two groups and treated as follows: (a) untreated (*n* = 10); (b) methyl mercury chloride treated (CH_3_HgCl_2_) (5 mg/kg,* po*., *n* = 10) [12]. The experiments were performed 24 hrs after treatment.

### 2.2. Biochemical Studies

Malondialdehyde (MDA) a stable product of lipid peroxidation, which is generally used as an index of free radical production was determined by reaction with thiobarbituric acid (TBA) [13]. Estimation of NO was done indirectly by measuring the serum levels of the products of NO metabolism, as recovery of nitrite and nitrate from serum. Nitrites and nitrates (NOx) were measured using the Griess Reagent and the resultant purple azo derivative was measured spectrophotometrically at an absorbance of 540 nm [14].

### 2.3. Systolic Blood Pressure

Blood pressure was measured in rats by noninvasive blood pressure (NIBP) technique using the IN125/R NIBP System which was used in conjunction with a PowerLab system to obtain non-invasive blood pressure measurement from rats (with tail diameters of 5–10 mm). NIBP uses a specialized tail cuff and pulse transducer to intermittently measure blood pressure based on the periodic occlusion of tail blood flow [15]. 

### 2.4. Vasoreactivity Experiments

Thoracic aorta was carefully dissected out from rat and cleaned of connective tissue of both groups. For reactivity experiments, the thoracic aorta was divided into segments that were 2 mm in length. For isometric tension recording, aortic rings were mounted in an organ bath, between a stationary stainless steel hook and an isometric force transducer (Grass FT-03, USA), and changes in isometric tension were recorded by a PowerLab data acquisition system (8SP 20B, AD Instruments, Australia) with a computerized analysis programme (Chart 5.4.2, AD Instruments, Australia). Vessels were maintained at 37°C in an organ bath containing 10 mL of modified Krebs-bicarbonate buffer solution of the following composition (in mM): Nacl 118; KCl 4.8; MgSO_4_ 1.2; KH_2_PO_4_ 1.2; NaHCO_3_ 2.5; CaCl_2_ 2.5; and glucose 11.0; pH, 7.4, bubbled with 95% O_2_ and 5% CO_2_. Aortic segments were subjected to a tension of 2 g that was readjusted every 15 min during a 60 min equilibration period before drug administration. Vessels were initially exposed to 75 mM KCl to check their functional integrity, and the presence of endothelium was confirmed by the ability of ACh (10 *μ*M) to relax segments precontracted with phenylephrine (PE) at a concentration that produces close to 50% of the contraction induced by 75 mM KCl [16]. 

To evaluate the relaxation, dependent on and independent of the endothelium, concentration-response curves were performed with ACh (10^−12^ to 10^−4^ M), isoproterenol (IP, 10^−12^ to 10^−4^ M), and sodium nitroprusside (SNP, 10^−12^ to 10^−4^ M), respectively in endothelium intact and endothelium denuded vessels in aorta previously contracted with PE. Endothelium was removed by rubbing the lumen with forceps. Response to ACh (10 *μ*M) was observed to confirm denudation (no vasorelaxation) which was validated by histology.

The effects of L-NAME 100 *μ*M glybenclamide (10 *μ*M), SOD (10 U/mL) plus catalase (100 U/mL), and SOD + catalase along with L-NAME were investigated separately in endothelium intact vessels to evaluate the participation of NO, EDHF and ROS in ACh responses.

### 2.5. Data Analysis and Statistics

All values are expressed as mean ± SEM of the number of animals used in each experiment. In the vascular reactivity experiments, vasodilator responses were expressed as the % change of the previous contraction to PE. To compare the effect of L-NAME, glybenclamide, SOD + catalase, and SOD + catalase along with L-NAME on the response to ACh in segments from the untreated and methyl-mercury-chloride-treated groups; the results were expressed as % change in response to ACh (10 *μ*M). The results were analyzed using Student's *t*-test for comparison between groups. Differences were considered statistically significant at *P* < 0.05.

## 3. Results

No differences in body weight were observed between the two groups due to treatment with methyl-mercury-chloride exposed rats, as previously described [17, 18].

### 3.1. Effect of Methyl Mercury Chloride on Systolic Blood Pressure (SBP)

**Figure 1:**
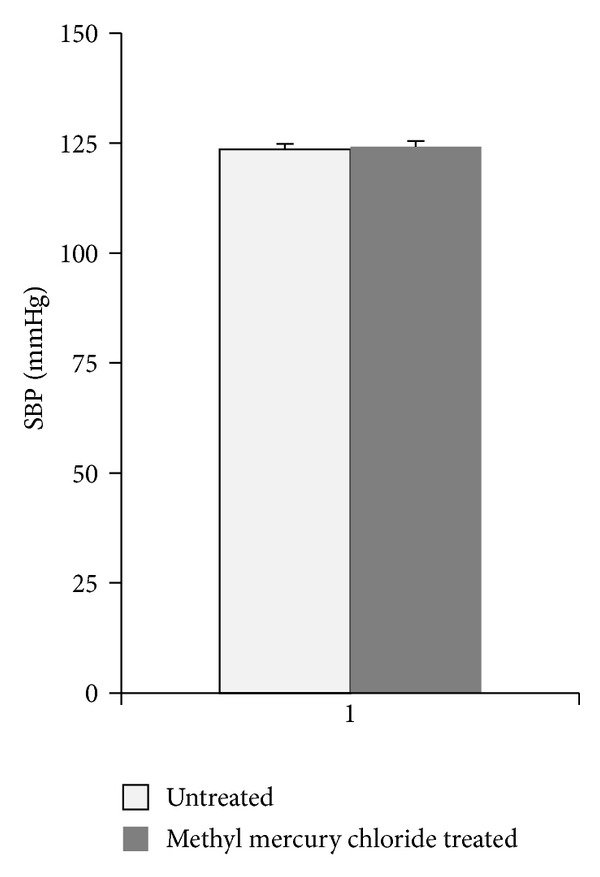
Effect of methyl mercury chloride treatment on systolic blood pressure (SBP). Mean values of systolic SBP in the untreated rats (*n* = 10) or rats treated with methyl mercury chloride (*n* = 10).

Acute treatment with methyl mercury chloride did not change the SBP in the exposed rats (Figure 1). 

### 3.2. Methyl Mercury Chloride Treatment Modulates Endothelium-Dependent Vasodilator Responses via NO Pathway

**Figure 2:**
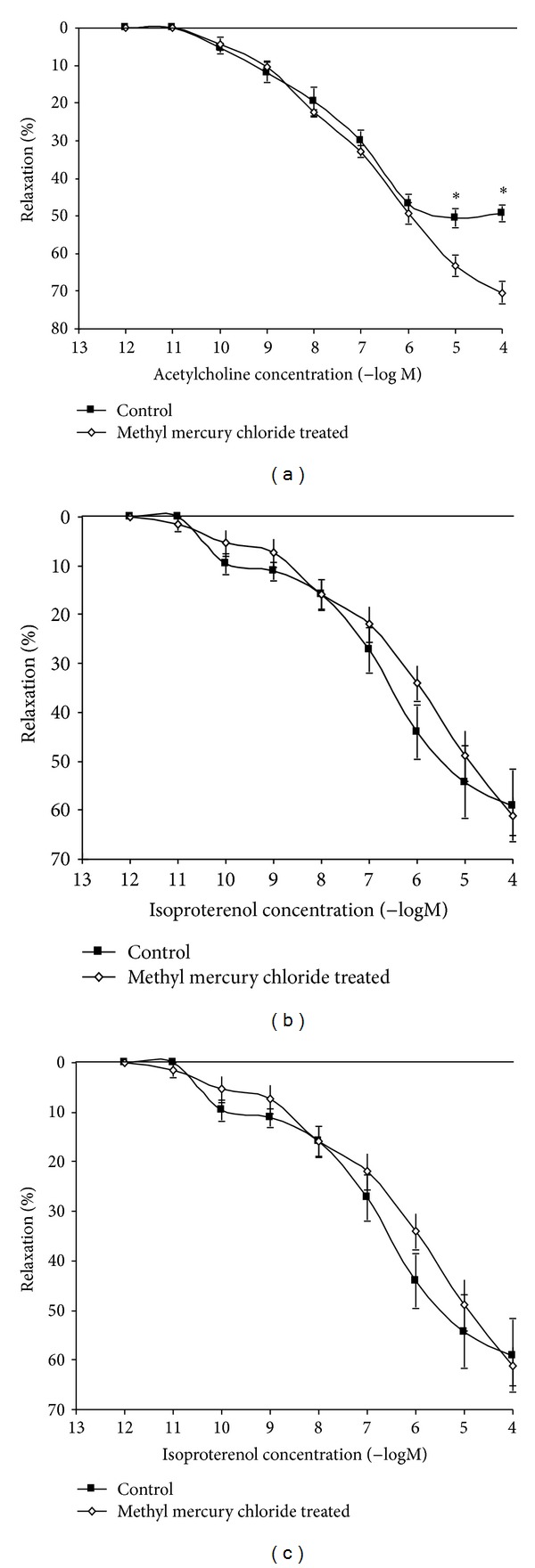
Effect of methyl mercury chloride treatment on the vascular relaxation response to acetylcholine, isoproterenol, and sodium nitroprusside. Concentration-response curves to (a) acetylcholine (ACh), (b) isoproterenol (IP) and (c) sodium nitroprusside (SNP) in the aortas of rats untreated, treated with methyl mercury chloride (*n* = 10) precontracted with PE. The results (mean ± SEM) are expressed as percentage of the response to PE. *t*-test, *P < 0.05.

Exposure to ACh, IP, and SNP produced concentration-dependent relaxation in the aortic rings from both groups. Acute exposure to methyl mercury chloride increased the vascular response to ACh, significantly (Figure 2(a), Table 1). IP, and SNP responses were similar in untreated as well as methyl-mercury-chloride -treated animals (Figures 2(b) and 2(c), Table 1). Responses in endothelium-denuded aortic rings were similar to ACh IP, and SNP in untreated as well as methyl-mercury-chloride treated rats (Table 2). These results suggest that acute exposure to methyl mercury chloride modulates the endothelium dependent vascular responses without affecting the endothelium-independent mechanisms.

**Figure 3:**
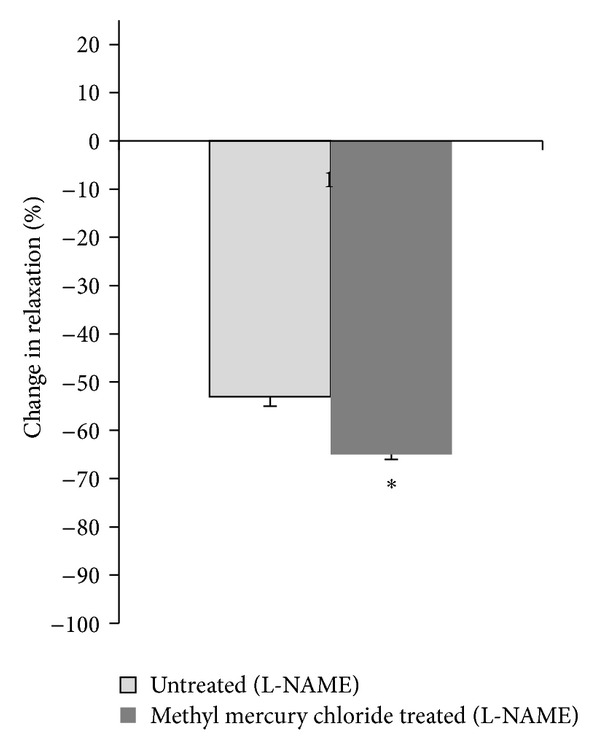
Effect of methyl mercury chloride treatment on NO modulation of the vasodilator response to ACh. % change in ACh (10 *μ*M) response curve in aortic segments precontracted with PE in the presence of L-NAME in the two experimental groups. *P < 0.05.

**Figure 4:**
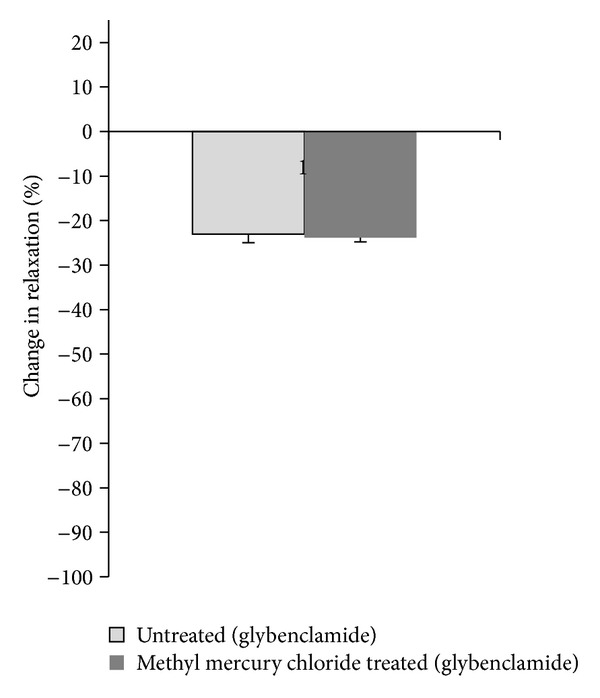
Effect of methyl mercury chloride treatment on EDHF modulation of the vasodilator response to ACh. % change in ACh (10 *μ*M) response curve in aortic segments precontracted with PE in the presence of glybenclamide in the two experimental groups. *P < 0.05.

To verify that the endothelial modulation in the vascular response to ACh in methyl-mercury-chloride-exposed rats was due to alterations in the effects of NO or EDHF, we used the NOS inhibitor L-NAME (100 *μ*M) and K_ATP_ channel inhibitor glybenclamide (10 *μ*M) *in vitro*. Both of the drugs decreased the response to ACh in aortic rings from both untreated and methyl-mercury-chloride-treated rats. However, the % change in values for L-NAME show that this decrease was significantly increased in rats treated with methyl mercury chloride when compared to untreated rats (Figure 3). The % change in values for glybenclamide was similar in treated and untreated rats (Figure 4). 

#### 3.2.1. SOD and Catalase Reduce the Increased Participation of ROS in Depressing the Vasodilator Responses of Methyl Mercury Chloride Treated Rats

**Figure 5:**
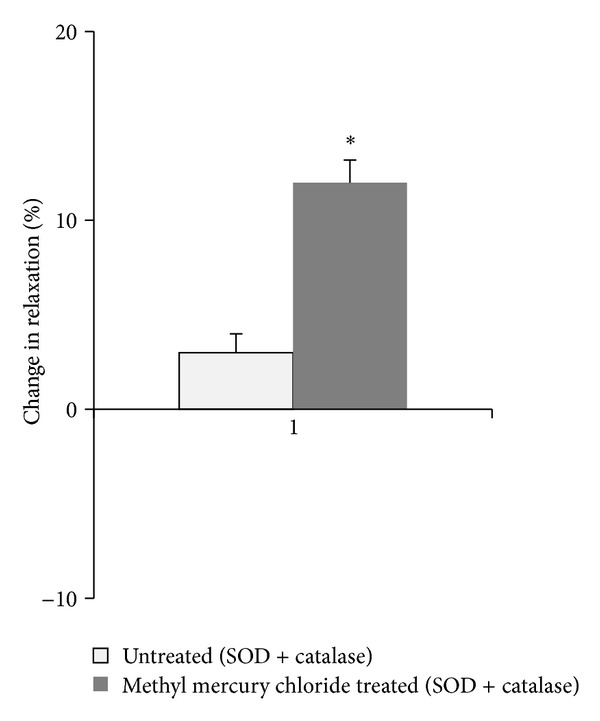
Effect of methyl mercury chloride treatment on ROS modulation of the vasodilator response to ACh. % change in ACh (10 *μ*M) response in aortic segments precontracted with PE in the presence of SOD + catalase in the two experimental groups. *P < 0.05.

**Figure 6:**
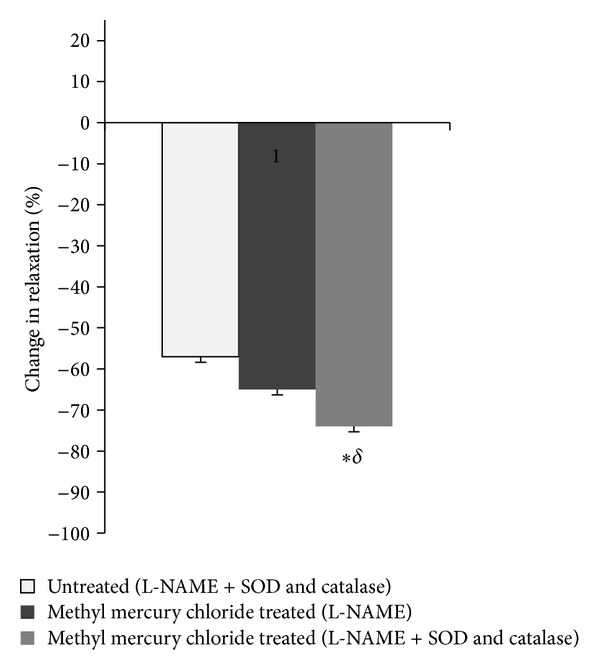
Effect of methyl mercury chloride treatment on ROS modulation of NO-mediated vasodilator response to ACh. % change in Ach (10 *μ*M) response curve in aortic segments precontracted with phenylephrine in the presence and the absence of SOD + catalase along with L-NAME in the two experimental groups. *P < 0.05, compared with the response to ACh in the presence of L-NAME + SOD and catalase from untreated group. ^§^ *P* < 0.05, compared with the response to ACh in the presence of L-NAME + SOD and catalase in methyl-mercury-chloride-treated group (L-NAME).

To evaluate the contribution of oxidative stress in the vascular alterations induced by methyl mercury chloride, we assessed the role of superoxide anion in ACh responses *in vitro* using SOD + catalase. This drug significantly increased the ACh responses in methyl-mercury-chloride-treated rats (Figure 5). To investigate whether ROS play any role in enhanced NO-mediated vasorelaxation induced by methyl mercury chloride, we used SOD + catalase along with L-NAME. These drugs reduced the ACh responses in both untreated and methyl-mercury-chloride-treated rats. However the decrease was significantly more in methyl-mercury-chloride-treated-rats (Figure 6).

### 3.3. Effect of Methyl Mercury Chloride on Lipid Peroxidation and NO in Plasma

**Figure 7:**
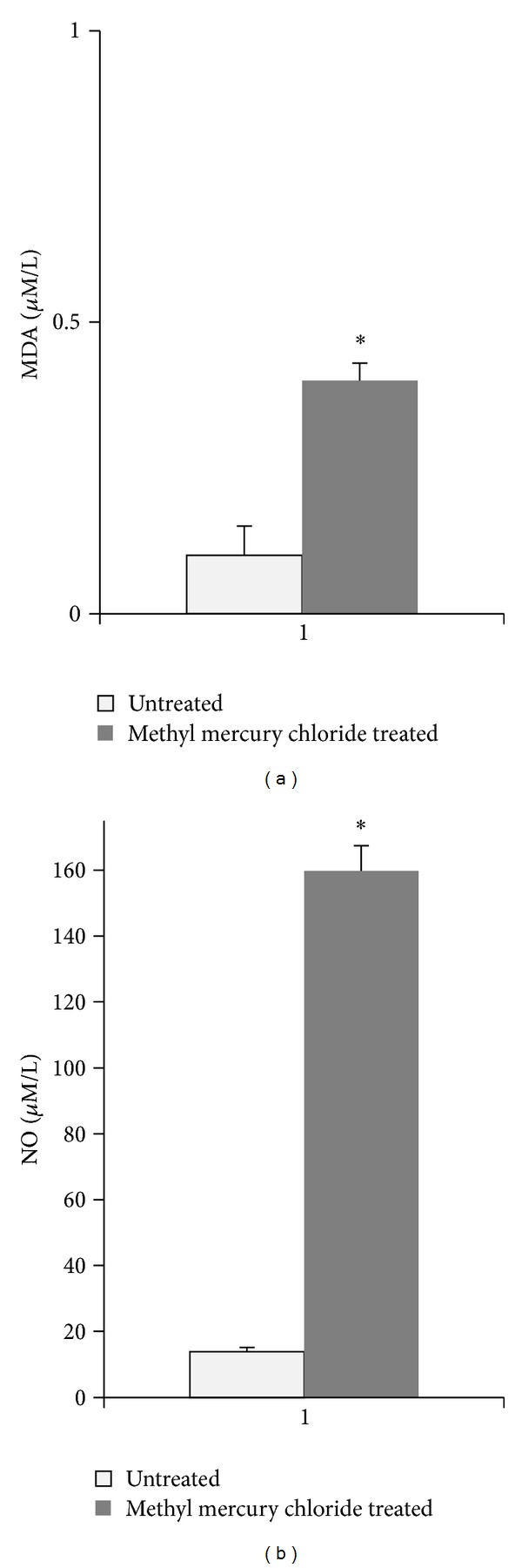
Effect of methyl mercury chloride treatment on MDA (a) and NO (b) in plasma levels of untreated and treated rats with methyl mercury chloride (mean ± SEM; *n* = 10). *P < 0.05.

Acute treatment with methyl mercury chloride significantly increased in oxidative stress and lipid peroxidation, as demonstrated by increased plasma MDA levels (Figure 7(a)). Methyl mercury chloride treatment also significantly increased the plasma NO levels (Figure 7(b)). 

## 4. Discussion

The present study demonstrates that methyl mercury chloride treatment (5 mg/kg; *po.*) significantly increases the NO-dependent vasodilator response in aorta of rats with no effect on SBP [18]. This effect is due to increased production of NO. It is interesting to note that this increase occurred even when there was increased MDA levels suggesting a state of redox imbalance and that these changes might be involved in the pathophysiology of the methylmercury toxicity. 

Mercury has been recognized as a risk factor for cardiovascular disease [1–3, 9]. Mercury is reported to significantly increase systolic and diastolic blood pressure [19, 20]. Recently, cardiovascular abnormalities attributed to parasympathetic dysfunction have been reported in a community exposed to mercury living near to an industrial complex in Korea [21]. A growing body of data now strongly indicates that damage to the blood vessel endothelium occurs following exposure to mercury, leading to a loss of endothelium-dependent vasorelaxation [7, 9] due to induction of oxidative stress [10]. Endothelial function of blood vessels is impaired in chronically mercury exposed rats as a consequence of the selective loss of NO-mediated relaxation [9].

In contrast, a cohort study, suggests that high blood mercury levels do not influence the vascular reactivity but increase the risk of hypertension [22]. Mercury *per se* is reported to induce an endothelial-dependent vasorelaxation and alter structure and function of vascular endothelial cells [7]. Mercury at 5 mg/kg, *iv* is reported to induce hypotension in rats [23].

In our study, we observed an increase in vascular reactivity to ACh in aortas of rats acutely exposed to methyl mercury chloride. Furthermore, we observed that endothelium removal abolished this effect, suggesting that methyl-mercury produces changes at the endothelial level without causing damage to the endothelium as previously reported [4–8]. The NO donor SNP induces relaxation by direct effect on the smooth muscle and via an endothelium-dependent pathway. Constitutive NOS (eNOS and nNOS), and not the iNOS, are the isoforms involved in the relaxation induced by SNP [24]. Response to SNP was not altered after methyl mercury chloride exposure suggesting that smooth muscle was not damaged by methyl mercury chloride exposure and that the alteration in eNOS and nNOS did not occur or was not enough to alter the response of SNP. In smooth muscle cells, *β*-adrenoceptor agonists, IP, promote vasorelaxation by activation of an adenylyl cyclase-cAMP-PKA pathway [25], and activation of *β*-adrenoceptors on the endothelium produces vasorelaxation that is mediated by the synthesis of NO [26]. As is the case in SNP, eNOS and nNOS are the isoforms involved in relaxation [27]. Response to IP was not altered after methyl mercury chloride exposure reaffirming our conclusion that endothelium dysfunction has not occurred and suggesting no or minimal alteration in eNOS and nNOS activity. 

ACh acts through muscarinic receptors present in endothelium. ACh enhances the release of NO via eNOS and EDHFs to produce vasorelaxation [28]. This finding is reinforced by the observed decrease in the endothelium-dependent vasodilator response induced by ACh in the presence of L-NAME and glybenclamide. Importantly in methyl-mercury-chloride-treated rats, treatment with L-NAME, a nonselective inhibitor of NOS prevents the increased response to ACh, suggesting that methyl mercury chloride acts mainly through the activation of NOS. More specifically, methyl mercury chloride increases NO availability, as demonstrated by the increased production of NO in methyl-mercury-chloride treated rats. Treatment with glybenclamide produced similar responses in untreated and treated rats suggesting that methyl mercury chloride does not alter the release of EDHFs.

An increase in the ACh response may be due to an increase in plasma NO levels following methyl mercury chloride exposure which may be overcoming the inhibitory effects of the free radicals [29, 30]. Increased plasma levels of NO may be due to increased activity of NOS. In a recent study, mercury induced NF-*κ*B activation this result in the increased expression of COX-2 and iNOS [31]. An increase in iNOS may lead to over production of NO which is an important factor in tissue trauma. Increases in NO level may be the first stage of the toxic oxidative reaction that is harmful for the tissues [32]. The iNOS expression has been reported to attenuate the ACh response [33]. However, in the present study, we observed an increase in the response of ACh in methyl-mercury-chloride-exposed rats with an increase in NO. ACh-induced vasorelaxation is mediated predominantly by eNOS [34]. This implies that there is augmentation of NO production due to eNOS upregulation and not due to iNOS [35]. Increased production of NO is beneficial after arterial injury because of its positive effects on vasorelaxation, prevention of platelet aggregation, and regulation of endothelial cell migration [36]. However, the mechanisms that regulate eNOS expression and activity, responsible for NO production in vessels after methyl mercury chloride exposure, have to be studied.

SOD + catalase treatment further increased the ACh response in methyl-mercury-chloride-treated rats. The ability of SOD + catalase to further improve endothelial-dependent vasodilator responses, has been previously described in different hypertension models [37]. In addition, our group has previously demonstrated that when administered *in vitro* in the organ bath, SOD + catalase improved the impaired response to ACh in aortas from chronically mercury chloride-treated rats [9].

As expected, SOD + catalase treatment of rats prevented the increased participation of superoxide anion in the vasodilator response to ACh in the aortas of rats exposed to methyl mercury chloride. Previous studies have demonstrated that SOD + catalase reverses endothelial NO dysfunction in animals or humans with elevated levels of oxidative stress [9]. The role of oxidative stress is supported by the increased % change in response after treatment with SOD + catalase along with L-NAME suggesting that oxidative stress causes a selective loss of NO-mediated vasodilation.

In summary, our results demonstrate that the methyl mercury chloride induces an increase in the vascular relaxation to ACh in aorta of rats exposed to methyl mercury chloride without having any effect on SBP. This effect is becouse increased production of NO may be a result of stimulation of eNOS. In addition, we demonstrated that methyl mercury chloride does not affect the EDHF pathway. These results suggest that methyl mercury chloride induces NOS and increases the NO production along with inducing oxidative stress without affecting the EDHF pathway. 

## Figures and Tables

**Table 1:** Effect of methyl mercury chloride treatment of rats on maximum response (*R*_max⁡_) and sensitivity (pD2) to acetylcholine, sodium nitroprusside, and isoproterenol in endothelium intact aortic rings.

	ACh		SNP		IP	
	*R*_max⁡_	pD2	*R*_max⁡_	pD2	*R*_max⁡_	pD2
Untreated	52.7 ± 2.8	−7.4 ± 0.2	84.2 ± 2.8	−8.4 ± 0.1	59.0 ± 7.3	−7.7 ± 0.2
Methyl mercury chloride treated	70.4 ± 2.9*	−6.9 ± 0.1	84.4 ± 2.5	−8.2 ± 0.1	61.2 ± 4.0	−6.4 ± 0.3

Results are expressed as % of the previous contraction to phenylephrine and pD2 is expressed as −log one half; *P < 0.05 versus untreated.

**Table 2:** Effect of methyl mercury chloride treatment of rats on maximum response (*R*_max⁡_) and sensitivity (pD2) to acetylcholine, isoproterenol, and sodium nitroprusside in endothelium-denuded aortic rings.

	ACh		SNP		IP	
	*R*_max⁡_	pD2	*R*_max⁡_	pD2	*R*_max⁡_	pD2
Untreated	2.1 ± 1.3	6.1 ± 0.3	80.5 ± 3.5	8.1 ± 0.1	27.5 ± 2.8	7.9 ± 0.4
Methyl mercury chloride treated	3.3 ± 1.9	6.1 ± 0.02	80.5 ± 2.9	8.1 ± 0.2	27.5 ± 1.6	7.9 ± 0.7

Results are expressed as % of the previous contraction to phenylephrine and pD2 is expressed as −log one half.
